# Rehabilitation of a congenital palatal defect with a modified technique: a case report

**DOI:** 10.1186/1757-1626-1-39

**Published:** 2008-07-16

**Authors:** Bora Bagis, Elif Aydoğan, Ufuk Hasanreisoğlu

**Affiliations:** 1 Assistant Professor, Department of Prosthodontics, Faculty of Dentistry, Karadeniz Technical University, Trabzon, Turkey; 2 Research Assistant, Department of Prosthodontics, Faculty of Dentistry, Karadeniz Technical University, Trabzon, Turkey; 3 Professor, Department of Prosthodontics, Faculty of Dentistry, Ankara University, Ankara, Turkey

## Abstract

**Introduction:**

Constuction of the obturator prostheses and providing the patient's comfort with them are very difficult. Primary and the most important stage for these prostheses is to perform a proper impression.

**Case Presentation:**

A 55-year-old edentulous Turkish female patient with a congenitally maxillary defect was rehabilitated with an open hollow obturator prosthesis. After the preliminary impression was completed, a metal frame suitable with the maxillary defect was constructed manually and used for making the impression of the defect area. After the first part of the obturator was finished, second part which separates nasal cavity and oral cavity was constructed by the aid of the bulb.

**Conclusion:**

When constructing an obturator prosthesis, making a detailed impression from the defect area can be performed by the aid of a metal frame, and an intraorally shaped extension that separates oral cavity from nasal cavity might be more effective for adaptation of the prosthesis.

## Background

Maxillary defects can be sourced by congenital malformations or the acquired defects resulting from surgeries [1]. The maxillofacial-prosthodontist has two primary objectives for rehabilitation of the maxillary defects. These objectives can be explained as to restore the functions of mastication, deglutition, and speech and to achieve normal oro-facial appearance [2]. Defect of the maxilla, which occurs as a result of tumor surgery or congenitally, may be closed with an obturator, which is a disc or plate, natural or artificial. The prosthesis that restores the maxillary defect is termed as a maxillary obturator. [3]. The obturator prosthesis that close these defects and separate the oral and nasal cavities may be constructed in different sizes and shapes, depending on the extent of the defect [4]. Oburators are classified as solid, open hollow and closed hollow as to the nature of their extension into defect site [5-10]. An open or closed hollow obturator is usually preferred to reduce the weight of the prosthesis when the maxillary defect is large, [1]. Both open and closed hollow obturators allow for the fabrication of a lightweight prosthesis that can be tolerated by the patient while effectively extending into the defect. Closed hollow obturators are usually fabricated with acrylic resin (polymethyl metacrylate resin), which is porous and able to absorb water [11-13], so consequently the use of a closed hollow obturator is often avoided [6,9]. The reduced weight, better hygiene conditions, easy fabrication and increased speech ability are some of the advantages of using an open hollow obturator [14]. The retention of the maxillofacial prosthesis will vary with the size and configuration of the defect, the amount and contour of the remaining palatal shelf, height of the residual alveolar ridge, the size, contour, and lining mucosa of the defect and the availability of undercuts [1]. A stable and retentive obturator fabrication has some difficulties without the assistance of teeth or implants for additional support [15].

This clinical report describes the prosthetic rehabilitation of an edentulous patient with congenital maxillary defect with an obturator made by a modified technique to restore the defect and separate the oral and nasal cavities from each other.

## Case presentation

A 55-year-old edentulous Turkish female patient was referred to the Department of Prosthetic Dentistry in Karadeniz Technical University for examination and treatment. The patient had a history of congenital palatal defect with an opening between oral and nasal cavities. Detailed case history revealed that the oronasal opening was present since year of birth and the defect was not treated surgically. The patient's major complaint was being edentulous after looosing her natural teeth because of periodontal diseases. She had never had obturator prosthesis until she lost her last teeth. Construction of obturator prosthesis was decided for the rehabilitation of the patient after clinical examination.

**Figure 1:**
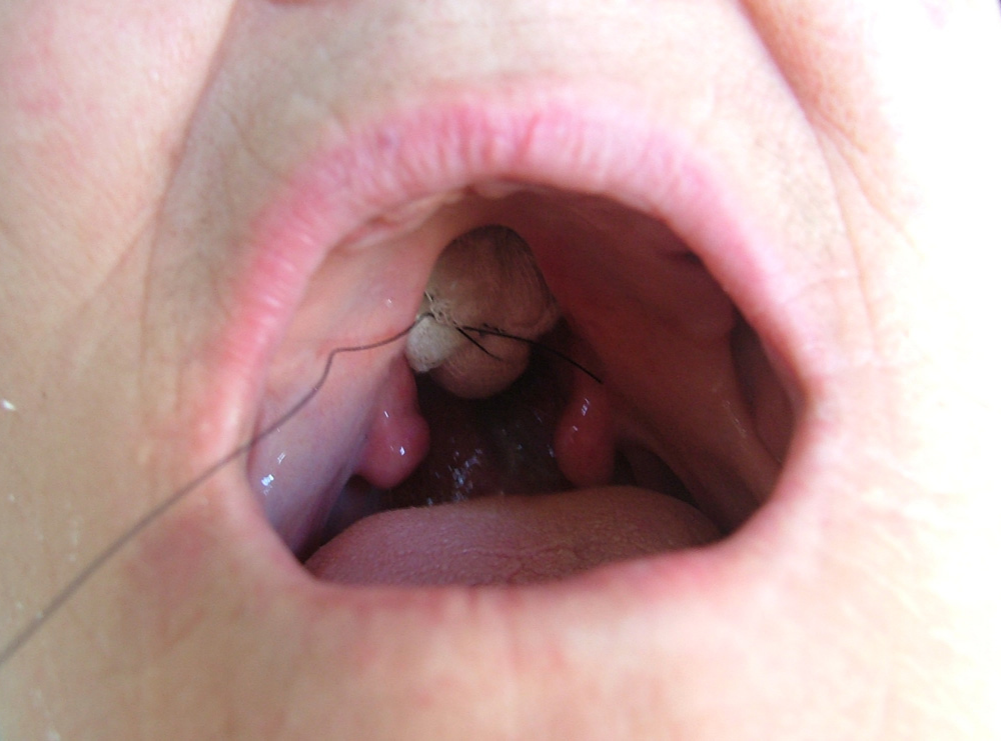
Intraoral view of the tampon inserted into maxillary defect.

**Figure 2:**
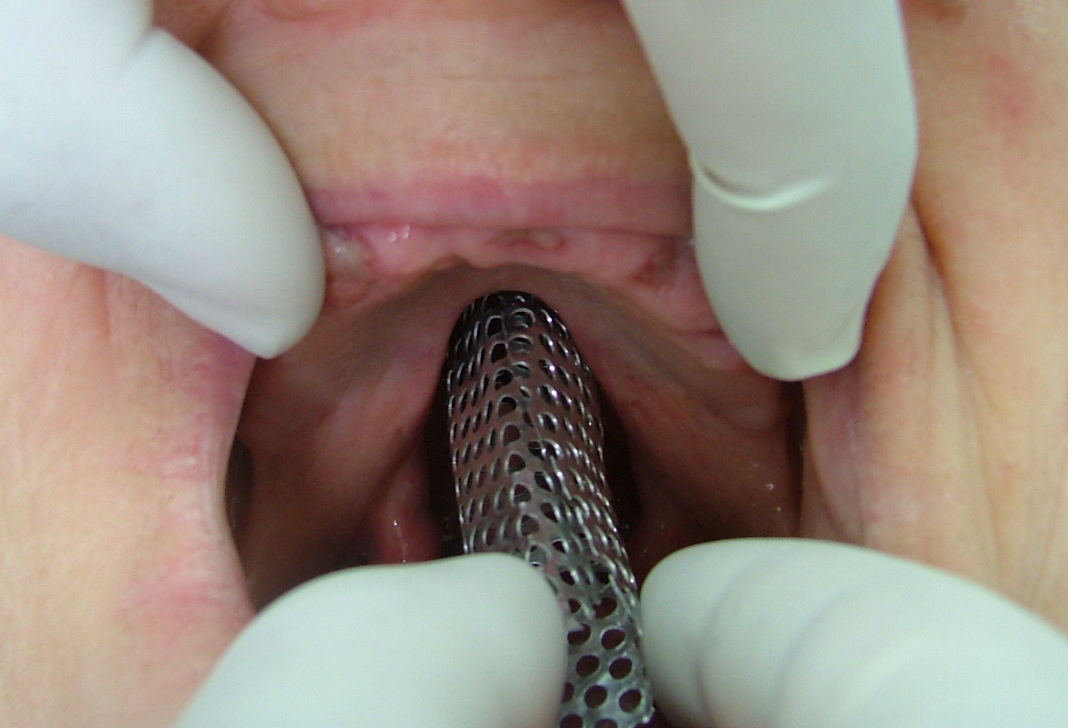
Metal frame shaped as to the size of the maxillary defect area.

**Figure 3:**
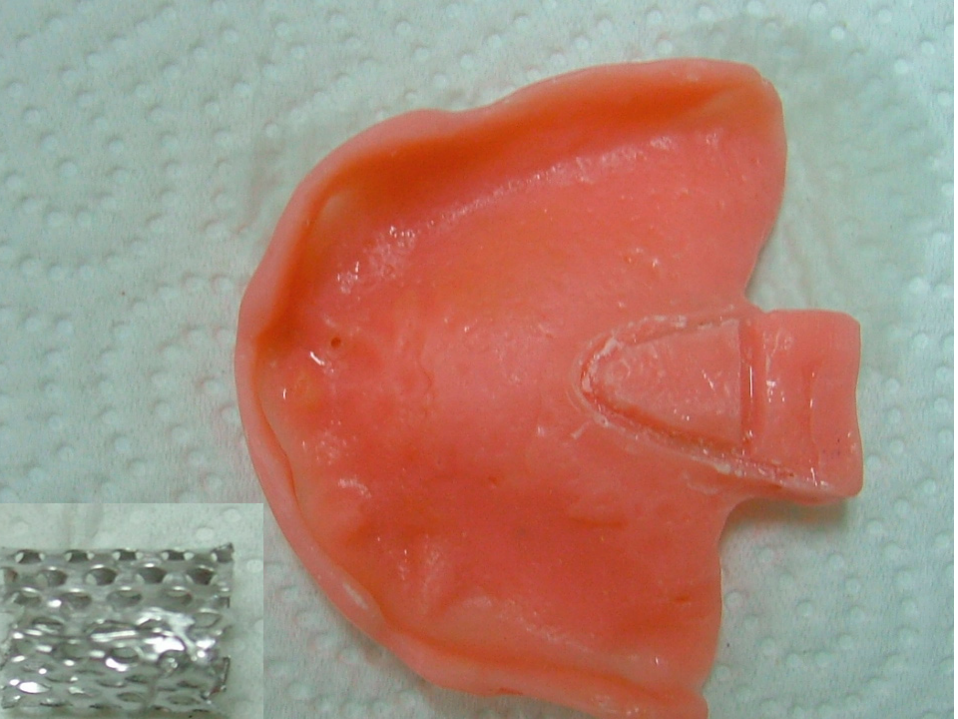
Metal frame tied with a metal cord and cavity drilled acrylic base.

**Figure 4:**
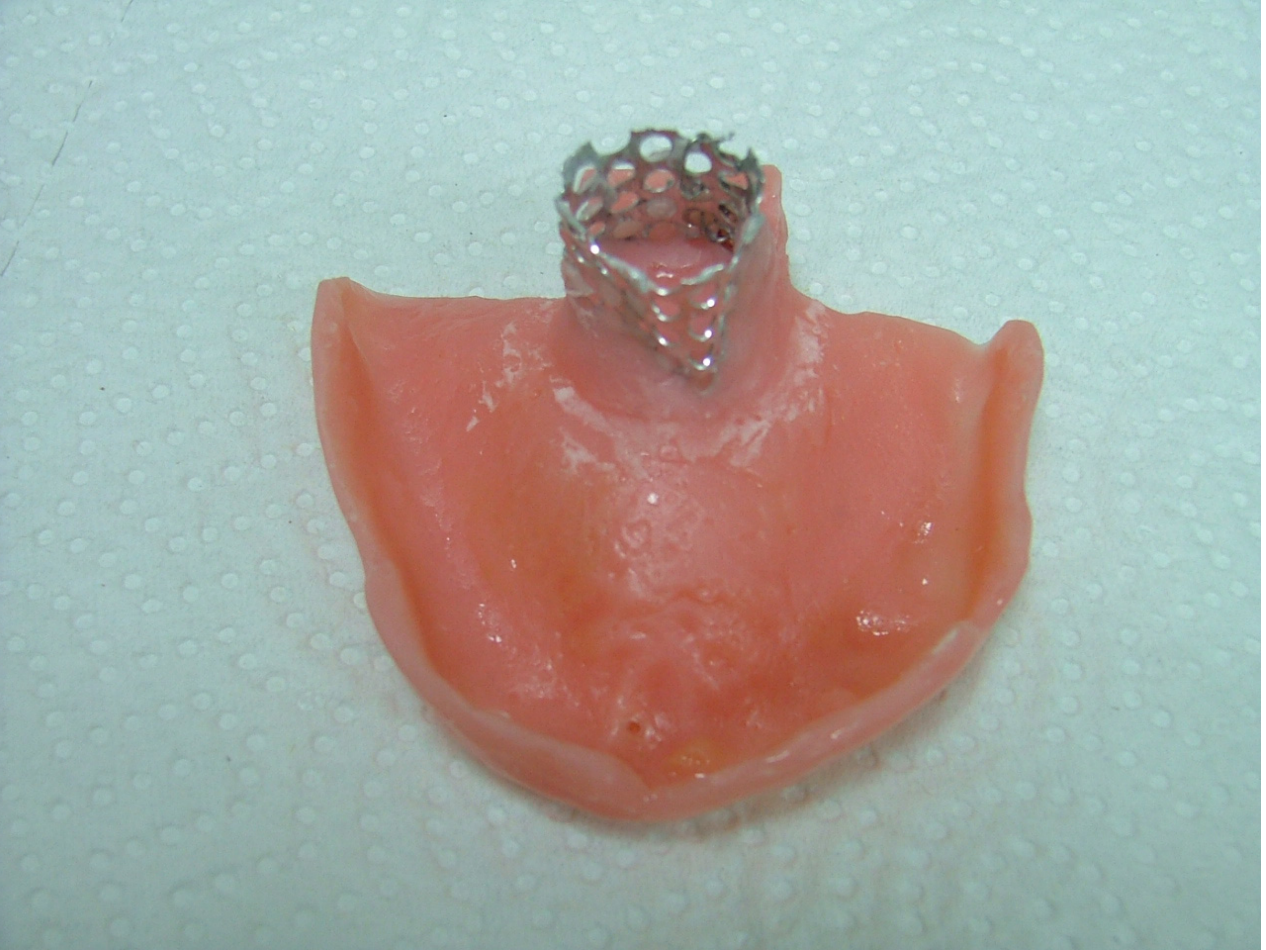
Metal frame molded with wax for optimum adaptation to the defect area.

**Figure 5:**
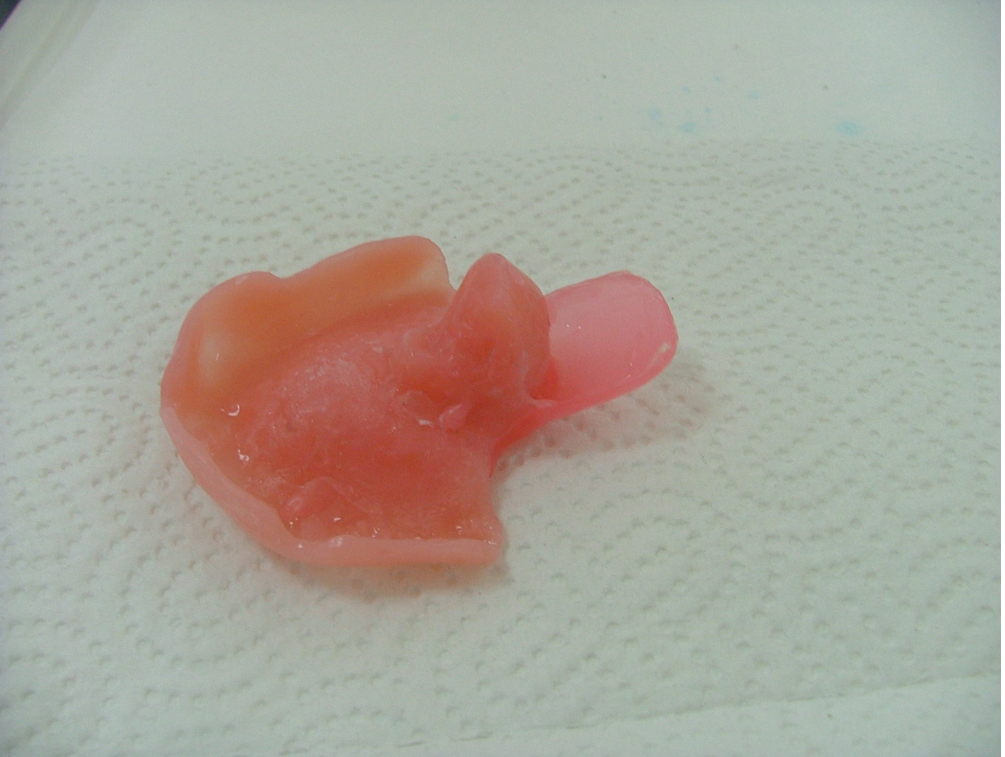
Shaping the second part of the bulb intraorally by a modeling wax.

**Figure 6:**
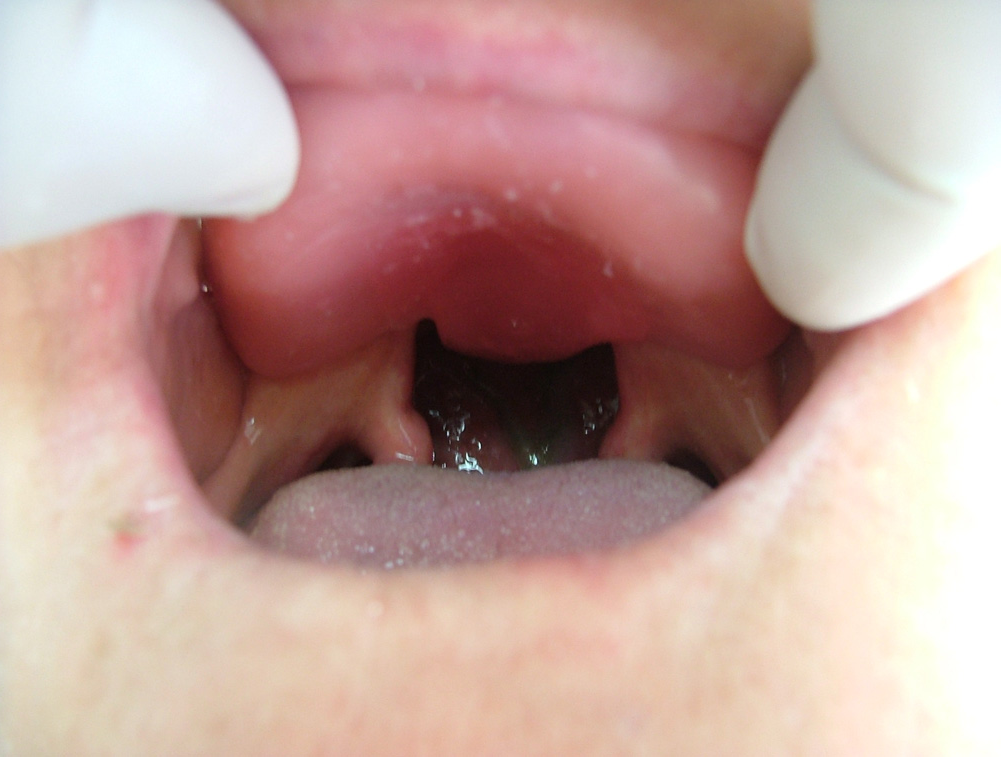
Adaptation of the acrylic base intraorally.

**Figure 7:**
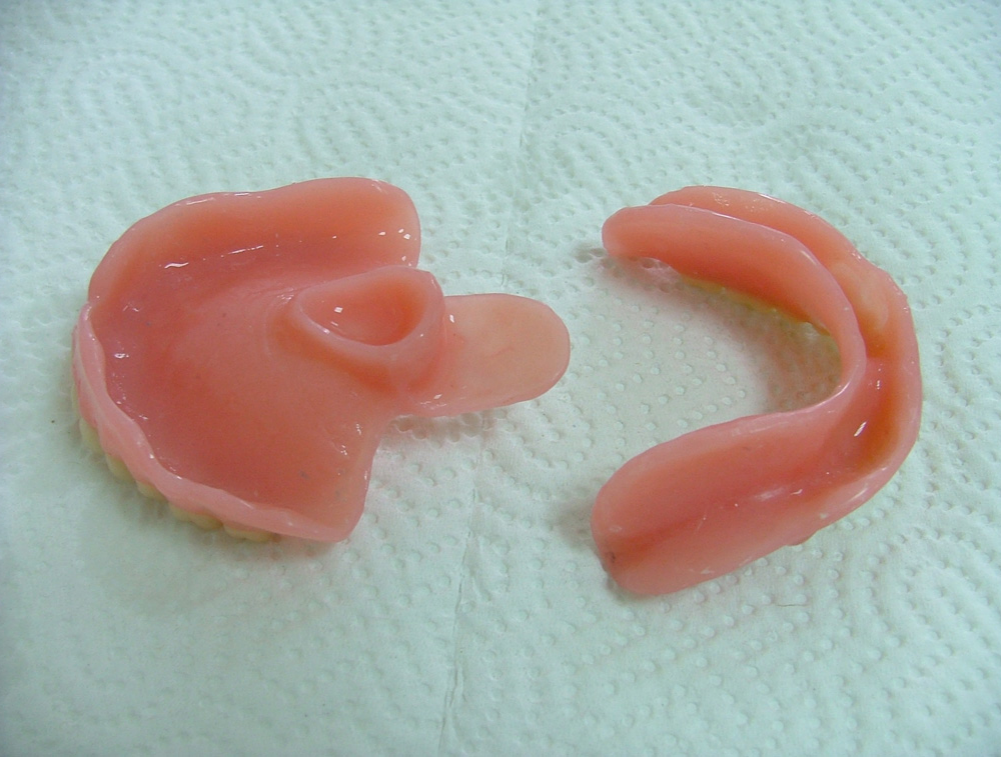
View of the final obturator prosthesis.

**Figure 8:**
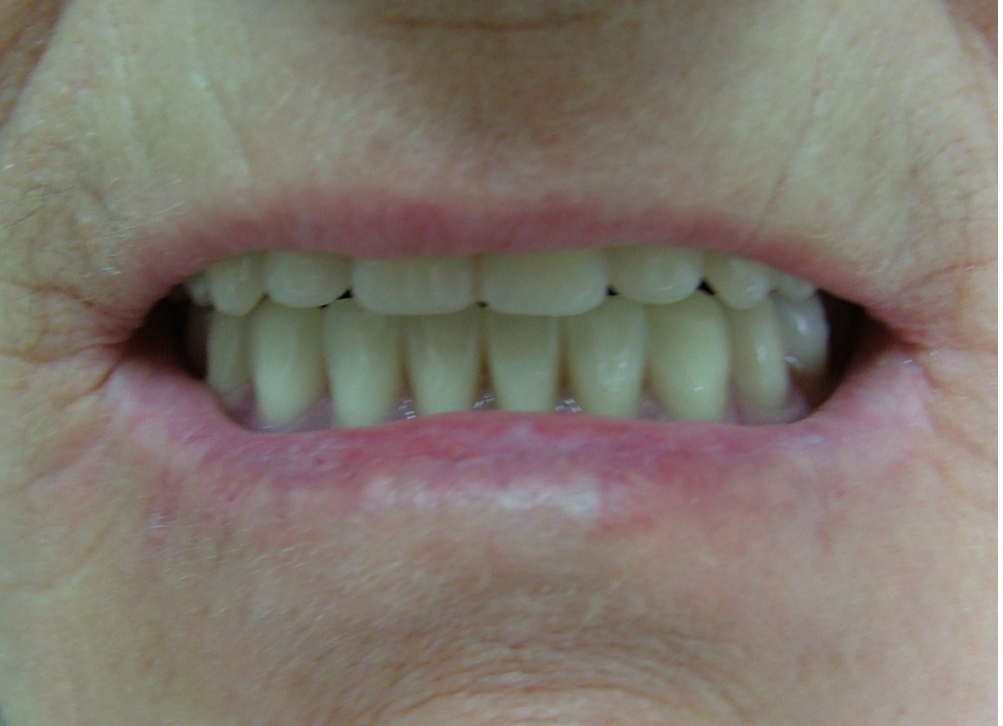
View of the obturator prosthesis in the mouth.

Local anesthetic spray (Xylocaine Pump Spray 10%, AstraZeneca, Sweden) was used for the palatal and post palatal region before making the impression. A tampon covered with Vaseline and anesthetic solution was placed in the defect cavity to protect the area from the residual impression material (Figure 1). The tampon was tied with a rope in order to take it out easily after the impression. Preliminary impression was made with an irreversible hydrocolloid and a diagnostic model was obtained by using type III dental stone. A poly methyl metachrylate resin base with a posterior extension was built up on this model. A metal frame was shaped as to the size of the maxillary defect area (Figure 2) It was tied with a metal cord and afterwards was suited to the cavity on the obturator's extended part, previously prepared with a bur (Figure 3). Then the metal frame was molded with a modeling wax to achieve adequate fit of the bulb to the defect cavity (Figure 4). The wax part was finished with acrylic resin. After the adaptation of the base to the denture bearing tissues was controlled, the second part of the bulb for separating the nasal and oral cavities from each other was constructed intraorally again using a modeling with wax (Figure 5). This wax was extended as much as the patient feels discomfort. Intraorally contoured wax part of the acrylic base was also fabricated with acrylic resin. The extended wax part was also fabricated with acrylic. Acrylic base was examined intraorally for inconvenience especially during the patient's functional movements like chewing, speaking or swallowing (Figure 6). Centric relation was determined with occlusal rims by using conventional maxillomandibular records. Acrylic teeth were arranged in a balanced articulation. After arranging the artificial teeth and reevaluating the occlusion, peripheral borders of the acrylic base was contoured with impression compound and final impression of the denture bearing tissues were made with zinc oxide eugenol impression paste. The obturator prosthesis was completed with conventional water bath polymerization technique (Figure 7, 8). Relining was performed with a silicone based soft relining material (Ufi Gel Permanent, Voco, Postfach Cuxhafen, Germany) to avoid tissue damage and to obtain better seal and retention. The patient was recalled next day, one week after and every third months for control. The major complaint of the patient was sore spots in the lower jaw and this was eliminated by relieving the respective tissue surface of the denture base. Soft relining material was changed every six months. The patient was satisfied with the prosthesis regarding function and phonation during the one-year control period.

## Discussion

The prosthetic rehabilitation of an edentulous patient with congenital maxillary defect with an obturator has some difficulties at the stages of impression and construction. Even the defect area is filled with a tampon before the impression, protecting the soft tissues from the residual impression materials and making a detailed impression is not easy. In this respect, recording the tissue borders with modeling wax around a metal frame intraorally may be more protective and still an effective method. Separating nasal and oral cavities from each other helps to form speech voices better and to protect nasal cavity from the food escape during chewing, and swallowing. Constructing a second part which extends to the palatal defect cavity on previously constructed bulb using an intraorally shaped modeling wax, helps the second part to cover the defect area without any damage to the soft tissues even during the functional movements. Fabricating method of this open hollow obturator provides better initial record base retention, which is also compatible with the oral tissues. And the design of this obturator prosthesis has also some advantages as: better phonation and protection of food escape to nasal cavity.

## Conclusion

Fabricating a successful obturator prosthesis used for the prosthetic rehabilitation of congenital or acquired defects in maxilla depends on making a detailed impression and constructing the prosthetic parts compatible with the oral tissues. This clinical report describes an intraoral technique for impression making and fabrication of open hollow obturator prosthesis.

## Competing interests

The authors declare that they have no competing interests.

## Authors' contributions

BB performed for the case and prepared the manuscript. EA helped at the some steps of constructing the prosthesis and to draft the manuscript. UH supervised the case and reviewed the manuscript. All authors read and approved the final manuscript.

## Consent

Written informed consent was obtained from the patient for publication of this case report and accompanying images. A copy of the written consent is available for review by the Editor-in-Chief of this journal.
